# Distinct gut microbiota and metabolome features of tissue-specific insulin resistance in overweight and obesity

**DOI:** 10.1080/19490976.2025.2501185

**Published:** 2025-05-07

**Authors:** Kelly M. Jardon, Alexander Umanets, Anouk Gijbels, Inez Trouwborst, Gabby B. Hul, Els Siebelink, Lars M.M. Vliex, Jacco J.A.J. Bastings, Rosa Argamasilla, Elodie Chenal, Koen Venema, Lydia A. Afman, Gijs H. Goossens, Ellen E. Blaak

**Affiliations:** a TiFN, Wageningen, The Netherlands; b Department of Human Biology, NUTRIM School of Nutrition and Translational Research in Metabolism, Maastricht University Medical Center+, Maastricht, The Netherlands; c Centre for Healthy Eating & Food Innovation, Maastricht University Campus Venlo, Venlo, The Netherlands; d Chair Group Youth Food and Health, Faculty of Science and Engineering, Maastricht University Campus Venlo, Venlo, The Netherlands; e Division of Human Nutrition and Health, Wageningen University & Research, Wageningen, The Netherlands; f DSM-Firmenich, Kaiseraugst, Switzerland

**Keywords:** Tissue-specific insulin resistance: obesity, gut microbiota, gut metabolites, GLP-1, metabolic subtyping, precision health

## Abstract

Insulin resistance (IR) is an early marker of cardiometabolic deterioration which may develop heterogeneously in key metabolic organs, including the liver (LIR) and skeletal muscle (MIR). This tissue-specific IR is characterized by distinct metabolic signatures, but the role of the gut microbiota in its etiology remains unclear. Here, we profiled the gut microbiota, its metabolites and the plasma metabolome in individuals with either a LIR or MIR phenotype (*n* = 233). We observed distinct microbial community structures LIR and MIR, and higher short-chain fatty acid (SCFA) producing bacteria, fecal SCFAs and branched-chain fatty acids and a higher postprandial plasma glucagon-like-peptide-1 response in LIR. In addition, we found variations in metabolome profiles and phenotype-specific associations between microbial taxa and functional metabolite groups. Overall, our study highlights association between gut microbiota and its metabolites composition with IR heterogeneity that can be targeted in precision-based strategies to improve cardiometabolic health. Clinicaltrials.gov registration: NCT03708419.

## Introduction

Insulin resistance (IR) is a key risk factor for the development of obesity-related diseases, including type 2 diabetes (T2D) and cardiovascular disease.^1^ Deterioration of cardiometabolic health can be prevented or delayed by lifestyle intervention, but there seems to be a large inter-individual variability in intervention response.^2,3^ Differences in environmental and endogenous factors such as age, sex, clinical characteristics, genetic susceptibility and the gut microbiota may underlie the variation in outcomes of lifestyle interventions.^4–6^ There is a critical need to better understand which modifiable factors should be targeted to achieve optimal health benefits in different subgroups of the population.

We and others have previously shown that there is substantial heterogeneity in the development of IR, with inter-individual differences in IR severity in metabolic organs including the liver and skeletal muscle.^7–10^ People with predominant liver IR (LIR) or predominant skeletal muscle IR (MIR) are characterized by pronounced differences in plasma metabolome, lipidome and inflammatory profiles, as well as the expression of inflammatory genes in abdominal subcutaneous adipose tissue.^7–9,11^ Furthermore, we recently showed in the PERSON study, a dietary intervention trial including 242 individuals with predominant MIR or LIR, that improvements of insulin sensitivity, glucose homeostasis, serum triacylglycerol and C-reactive protein in response to diets with varying macronutrient composition were dependent on this tissue-specific IR phenotype.^6^

Disturbances in the gut microbiota have been associated with obesity, T2D and alterations in host energy and substrate metabolism.^12–14^ Specific compositional changes and decreased microbial richness and diversity are commonly found in individuals with overweight or obesity.^15,16^ Furthermore, people with IR or T2D often show a decreased production of gut microbiota-derived short-chain fatty acids (SCFAs), including butyrate, acetate and propionate. These SCFAs are known for their beneficial effects on glucagon-like peptide-1 (GLP-1) secretion and host metabolism,^17–20^ differences in gut microbial composition may relate to distinct prediabetic phenotypes. Indeed, it has previously been shown that the overall gut microbiota composition is altered in individuals with impaired glucose tolerance (IGT, often characterized by MIR), while microbial composition of individuals with impaired fasting glucose (IFG, often linked to LIR) resembles more that of healthy individuals.^20^ Additionally, improvements in peripheral rather than hepatic insulin sensitivity were reported following both dietary fiber intervention and fecal microbiota transfer in people at cardiometabolic risk.^21,22^ Overall, various studies point toward the gut microbiota as significant marker of distinct cardiometabolic phenotypes and predictor of an individual’s postprandial glucose response and lifestyle intervention outcomes.^4,5,15,20,22–24^ Therefore, profiling the gut microbiota in MIR and LIR may contribute to a better understanding of the etiology of cardiometabolic disorders. In addition, considering the potential to reshape the composition and functionality of the gut microbiota by diet, it is an attractive target for novel precision nutrition strategies.^6,25^

Thus far, existing deeply-phenotyped cohorts investigating heterogeneity in tissue-specific IR did not include gut microbiota profiling. Considering the evident association between the gut microbiota and host metabolic health, we hypothesized that tissue-specific IR is characterized by distinct microbial signatures. In the present study, we therefore compared the composition of the gut microbiota and related gut metabolite profiles between well-phenotyped individuals with predominant MIR or LIR to gain a better understanding of the role of the gut microbiome in tissue-specific IR phenotypes.

## Methods

### Study design

The current cross-sectional analysis was part of the PERSON study (PERSonalized glucose Optimization through Nutritional intervention). The PERSON study is a 12-week intervention study with a randomized, parallel and double-blind design. In short, individuals with predominant LIR of MIR were included to follow either a low fat, high protein, high fiber diet (LFHP) or a diet high in mono-unsaturated fatty acids (HMUFA). Before and after the 12-week intervention, individuals underwent detailed metabolic and microbial phenotyping, as described in in previous work.^6,26,27^ Here, we performed a cross-sectional analysis on pre-intervention data at baseline. The Medical Ethical Committee of the MUMC+ approved the protocol (NL63768.068.17), which was registered at ClinicalTrials.gov (NCT03708419). Measurements were performed at the two main study centers at the Maastricht University Medical Center+ (MUMC+) and Wageningen University & Research (WUR) in the Netherlands, between May 2018 and November 2021. The study has been carried out according to the principles of the Declaration of Helsinki. All participants gave written informed consent prior to inclusion. All authors had access to the study data and reviewed and approved the final manuscript.

### Study participants

Out of 242 randomized participants in the PERSON study, we included *n* = 233 in the present cross-sectional study. Recruitment took place via advertisements in local (online) media, flyers and a volunteer database.^6,26,27^ Inclusion criteria were: participants aged 40–75 years, with a BMI of 25–40 kg/m^2^, body weight stability for at least 3 months (no weight gain or loss >3 kg), and a predominant LIR or MIR phenotype as determined by a 7-point oral glucose tolerance test (OGTT, *Assessment of tissue-specific insulin resistance*). All participants were Caucasian. Individuals were excluded from participation in case of pre-diagnosis of T2D, diseases or use of medication that affect glucose and/or lipid metabolism, antibiotics use 3 months prior to the study, major gastrointestinal diseases, history of major abdominal surgery, uncontrolled hypertension, smoking, alcohol consumption >14 units/week, and >4 h/week moderate-to-vigorous physical activity. Standardized protocols were used during a screening visit for assessment of compliance with in- and exclusion criteria.^6,26,27^ Participants who did not collect fecal samples according to the study protocol were excluded from analyses.

### Reference cohort with participants without tissue-specific IR

In addition to the 233 PERSON study participants, we included 15 participants from the CARMA study cohort (ClinicalTrials.gov: NCT04561284) and 15 participants from the SWEET study cohort (ClinicalTrials.gov: NCT04226911; WP3) who classified as ‘No MIR/LIR’ based on a 7-point OGTT, as described below, and from whom fecal samples were available. Briefly, these reference cohorts included adults (age range 18–65 years) with overweight or obesity (BMI range 25–35 kg/m^2^), selected in line with the in- and exclusion criteria of the main cohort. All fecal samples were collected at the MUMC+, Maastricht, the Netherlands, between August 2020 and October 2022.

### Assessment of tissue-specific insulin resistance and glucose metabolism

Plasma glucose and insulin values at fasting (*t* = 0 min) and *t* = 15, 30, 45, 60, 90, and 120 min during a 7-point OGTT (75 g glucose drink, Novolab, Geraardsbergen, Belgium) were used to determine tissue-specific IR.^6,26,27^ Enzymatic assays or ELISA were used to determine plasma glucose and insulin, respectively. Calculations for the hepatic insulin resistance index (HIRI) and muscle insulin sensitivity index (MISI) were used to estimate LIR and MIR.^28^ The MISI calculation has been optimized using the cubic spline method.^29^ Individuals were classified as ‘No MIR/LIR,’ ‘MIR,’ ‘LIR,’ or ‘combined MIR/LIR,’ using tertile cutoffs for MISI and HIRI based on a selected population of The Maastricht Study, which resembled the population of the PERSON study.^9,30^ From the main cohort, only isolated LIR and MIR phenotypes were included. Using the same cutoffs for MISI and HIRI, a ‘No IR’ group was included from the reference cohort and was only included in a sub-analysis.

The homeostasis model assessment of insulin resistance (HOMA-IR), HOMA of β-cell function (HOMA-β), Disposition Index and the Matsuda Index were calculated as previously described.^31,32^ Criteria of the World Health Organization (WHO) were used to define glucose status (normal glucose tolerance (NGT), impaired fasting glucose (IFG), impaired glucose tolerance (IGT) and T2D).^33^ The inflammatory marker C-reactive protein (CRP) was measured in fasting plasma with a Luminex immunoassay (DSM-Firmenich, Kaiseraugst, Switzerland).^6^

### Blood pressure

Systolic and diastolic blood pressure were measured in triplicate on the non-dominant arm with an automated sphygmomanometer after a 5-minute rest.

### Anthropometrics and body fat

Waist and hip circumference were measured in duplicate using a flexible measuring tape. Whole-body fat percentage was assessed using dual-energy X-ray absorptiometry (DXA) (WUR, Lunar Prodigy, GE Healthcare; MUMC+, Discovery A, Hologic). Visceral adipose tissue (VAT) volume was quantified at the MUMC+ only (3T MAGNETOM Prisma fit, Siemens Healthcare). Details of these methods have been described previously.^6^

### Habitual dietary intake and physical activity

Habitual dietary intake was assessed with a validated 163-item semi-quantitative food frequency questionnaire (FFQ).^34^ Goldberg’s method with the ratio of reported daily energy intake to estimated basal metabolic rate (BMR) was used to identify dietary misreporting. Underreporting (EI/BMR <0.87) and overreporting (EI/BMR >2.75) were excluding from analyses. Here, a total of *n* = 25 was excluded due to underreporting and *n* = 1 due to overreporting. Self-reported habitual physical activity was assessed with the Baecke questionnaire.^35^

### Plasma analyses

#### Plasma GLP-1

Plasma GLP-1 was determined in fasted condition (*t* = 0) and at *t* = 30, 60 90, 120 and 180 after ingesting a liquid high-fat mixed meal (HFMM, total 350 g containing 2.8 MJ, 49 g [64 en%] fat, 48 g [29 en%] CHO, 12 g [7 en%] protein).^27^ For analysis, 40 μl of dipeptidyl peptidase-IV inhibitor (Milipore Merck, Billerica, MA, USA) was added to EDTA and Aprotinin (Becton Dickinson, Eysins, Switzerland) tubes, respectively. Plasma samples were assayed for total GLP-1 immunoreactivity using an antiserum that reacts equally with intact GLP-1 and the primary (N-terminally truncated) metabolite as previously described.^36^ Analyses were performed in a subgroup of the main study cohort (*n* = 160) after random selection of samples.

#### Circulating SCFAs and BCFAs

For plasma (SCFA and branch-chain fatty acid (BCFA) concentrations, a fasting blood sample was collected in a 4 mL lithium heparin tube (BD, Plymouth, UK). Total plasma concentrations were measured using liquid chromatography – mass spectrometry (LC-MS). This liquid chromatography was performed using a micro flow ultra-high-performance liquid chromatography (UHPLC) instrument (Dionex Ultimate 3000) using protocols as described previously.^37,38^ Analyses were performed upon availability in a subgroup of the study cohort (MUMC+ study center, main cohort, *n* = 82).

#### Metabolome profiling

Fasting plasma metabolites were quantified using a targeted metabolomics approach based on the Biocrates MxP® Quant 500 kit (Biocrates Life Sciences AG, Innsbruck, Austria), and 630 metabolites from 26 compound classes were profiled. Samples were processed according to the manufacturer’s instructions. Analysis of sample extracts as well as of reference standards and quality controls was carried out by UHPLC and flow injection analysis, both coupled to tandem mass spectrometry. An Agilent 1290 series UHPLC system coupled to a 6500 + qTrap mass spectrometer equipped with an Ion-Drive Turbo V® ESI source (both Sciex, Foster City, CA, USA) was used for the analysis. Chromatographic and mass spectrometric parameters were set as indicated by the manufacturer of the kit. Data analysis was carried out in the Biocrates MetIDQ software.^39^ For quality control purposes, all potential metabolites were inspected and excluded when 75% or more of the analyzed samples lacked detectable signal.

### Fecal analyses

#### Bristol stool scale

Participants documented their stool consistency using the Bristol stool scale (BSS) at the time of feces collection, as a rough estimator of colonic transit.^40^ The stool type was rated on a scale of 1 to 7, with types 1 and 2 associated with constipation and types 6 and 7 associated with diarrhea.^41^

#### Fecal sample collection

Fecal samples were collected at home and stored for a maximum of 24 hours in the participants’ freezer at − 20°C. Upon arrival in the lab, the samples were stored at −80°C prior to analysis. Samples were kept frozen during transport from collection to storage and from storage to analysis.

#### Fecal SCFAs and BCFAs

Fecal SCFAs and BCFAs were measured using ion exchange chromatography with conductivity detection (Brightlabs, Venlo, The Netherlands). Fecal matter was centrifuged at 14,000 rpm for 10 min, filtered through a 0.45 μm PFTE filter, and diluted in the mobile phase (1.5 mm aqueous sulfuric acid).^42^ An automatic sampler 730 (Metrohm, Herisa, Switzerland) was used to load 10 ml of sample to the column, and the compounds were eluted based on their pKa. Ion exclusion chromatography (IEC) was used for analysis, using an 883 chromatograph (IC, Metrohm,) equipped with a Transgenomic IC Sep ICE-ION-300 column (30 cm × 7.8 mm × 7 μm) and a MetroSep RP2 Guard. Column flow was set at 0.4 mL/min and temperature at 65 °C. Suppressed conductivity detection ensured proper detection of SCFA and BCFA. Analyses were performed in a subgroup of the main study cohort (MUMC+ study center, *n* = 117).

#### Microbiota analysis

Amplicon sequencing of the 16S rRNA gene V3-V4 region was performed to determine microbiota composition as reported before.^43,44^ In short, QIAamp Fast DNA Stool Mini Kits (Qiagen Benelux, Venlo, the Netherlands) were used for genomic DNA isolation. A 2-step PCR was used to generate barcoded amplicons from the V3-V4 region of 16S rRNA genes (341F: 5’-CCTACGGGNGGCWGCAG-3’; 785 R: 5’-GACTACHVGGGTATCTAATCC-3’). 10–25 ng genomic DNA was used as template for the first PCR with a total volume of 50 μl. The second PCR was used to combined purified PCR products with sample-specific barcoded primers (Nextera XT index kit, Illumina, San Diego, CA, USA). After each PCR step the amplicons were purified (QIAquick PCR Purification Kit) followed by amplicons size (Fragment analyzer, Advanced Analytical, Ankeny, US) and concentration (fluorometric analysis, Qubit™ dsDNA HS Assay Kit) assessment. Purified amplicons were pulled equimolarly and sequenced using the paired-end (2×) 300 bp MiSeq protocol (Illumina, Eindhoven, The Netherlands). The Illumina CASAVA pipeline (v1.8.3) was used for raw data analysis and demultiplexing based on sample-specific barcodes. Quantitative Insights Into Microbial Ecology 2 (QIIME2) software was used for initial microbial analyses.^45^ Reads were imported as QIIME2 artifacts and the dada2 plugin was used for de-nosing and construction of amplicon variant sequencing (ASV) table.^46^ The taxonomic assignment of ASVs was performed with pre-fitted Scikit-learn based Naive Bayes classifier trained on full length 16s rRNA gene sequences from the SILVA v138 database.^47^ A phylogenetic tree of the ASVs representative sequences was inferred from MAFFT sequence alignment. Samples having low reads after processing (<5000 reads; minimal count was 5427) were excluded.

### Statistical analyses

#### Clinical characteristics

Baseline characteristics were compared between LIR and MIR phenotypes using Student’s independent T-test for normally distributed numerical data, Mann-Whitney test for non-normally distributed numerical data, and Fisher’s exact test for categorial data. For comparison of a subset of baseline characteristics between 3 phenotypes (no IR, LIR and MIR), differences were tested using respectively one-way ANOVA and Kruskal-Wallis tests with post hoc Bonferroni testing. Two-tailed *P* < 0.05 was considered statistically significant. Differences in plasma and fecal gut metabolites were tested using linear Mixed-Effect models implemented in the lme4^48^ package, and lmerTest^49^ as used for term significance estimation. Variable Sex was used as random variables and Phenotype, Age, BSS, Energy Intake, and Fibers Intake were used as fixed variables (*y ~ Phenotype + Age + BSS + Energy Intake + Fiber Intake + (1|Center) + (1|Sex)*). Fecal metabolite concentrations were log transformed (log_10_) to improve normality. Here, FDR-adjusted *Q*-values of > 0.05 were considered statistically significant.

#### Gut microbiota

All downstream microbiota and metabolite-related analyses were performed using the R v4.1.2 programming language and environment.^50^ Using the qiime2R package,^51^ we imported the ASV table, rooted phylogenetic tree, taxonomic table, and supporting data, and combined them into a phyloseq object.^52^ Prior to further analysis, ASVs with fewer than 10 reads across all samples, not assigned to any bacterial phylum, and assigned to chloroplasts or mitochondria were removed. Taxonomic names were shortened to the last assigned taxonomic level and made unique by the addition of unique identifier.

##### Alpha diversity

The total number of observed taxa (Observed), Shannon, Inverted Simpson (InvSimpson), and Phylogenetic Diversity (PhyloDiversity) diversity indexes were used to assess microbial richness as implemented in the phyloseq^52^ and picante^53^ packages. Prior to diversity calculation, the ASV count per sample was normalized by rarefaction (phyloseq[]) at a depth of 5427 reads. To assess differences in bacterial diversity between individuals with different phenotypes, we used linear Mixed-Effect models implemented in the lme4^48^ package, and lmerTest^49^ as used for term significance estimation. Variables Center and Sex were used as random variables and Phenotype, Age, BSS, Energy Intake, and Fibers Intake were used as fixed variables (*y ~ Phenotype + Age + BSS + Energy Intake + Fiber Intake + (1|Center) + (1|Sex)*). Phylogenetic Diversity and number of observed were without transformation as dependent variables, and Inverted Simpson and Shannon indexes were transformed by extracting square root and taking exponents of two respectively. Here and in following analysis all continuous variables were z-transformed prior to model fitting. In the model that utilized the data without the control group, Center and Sex were used as random effects and when the No IR group was included only Sex was included. Here, *P* < 0.05 was considered statistically significant.

##### Beta diversity

Differences in overall microbial composition were investigated by calculating dissimilarity distances between samples at the ASV taxonomic level, followed by testing the strength and significance of grouping with Permutational Multivariate Analysis of Variance Using Distance Matrices (adonis2) as implemented in the vegan package.^54^ Prior to dissimilarity distance calculations, ASV count was normalized with the Cumulative-Sum Scaling (CSS) as implemented in the metagenomeSeq package.^55^ Dissimilarity distances (weighted and unweighted UniFrac, Jaccard, Bray – Curtis) were calculated using the phyloseq package. The following model *Distance Matrix ~ Age + BSS + Energy Intake + Fiber Intake + Center + Sex + Phenotype* with 999 unconstrained permutation was used for adonis2 analysis. This analysis allowed to assess the significance (*P* < 0.05) and effect size of the included independent variables on overall microbial composition. Dissimilarities in the overall bacterial composition were visualized by creating Distance-based Redundancy Analysis (dbRDA) ordination as implemented in the vegan package, followed by visualization with a custom R function. A model identical to that used for adonis2 was employed to construct dbRDA.

##### Taxa differential abundance

Taxa differentially abundant (DA) between different phenotypes was identified using Zero-Inflated Negative Binomial Regression (ZINB) as implemented in Maaslin2 package. The Phenotype, Age, BSS, Energy Intake, and Fibers Intake were used as fixed effects and Center and Sex were used as random effects. DA was tested at the ASV and Genus taxonomic levels, and only taxa with 25% or more prevalence in a group were used in the analysis. Prior models building Trimmed Mean of *M* values (TMM) normalization and no transformation (NONE) of taxa count were applied as implemented in Maaslin2 function. The resulting *P*-values were adjusted using Benjamini and Hochberg (BH) adjustment method. *Q* < 0.1 was considered statistically significant.

##### Metabolomics

Group differences between IR phenotypes in individual metabolites were tested using the LMM following the same procedure as described above for the alpha diversity statistical analysis. Retrieved P-values were adjusted using the Benjamini-Hochberg FDR (stats package) per metabolite type. The overall group differences in metabolite composition were tested by performing Constrained Correspondence Analysis (CCA), as implemented in the vegan package. Phenotype, Energy, Intake, Age, BSS, Fibers Intake, Center, and Sex were used as constraining variable (*metabolites ~ Phenotype + Energy Intake + Age + BSS + Fibers Intake + Center + Sex*). The constrains significance was assessed using a permutation test (anova.cca), as implemented in the vegan package. Prior to CCA and LMM modeling to metabolite concentrations pseudo count (one) was added with following log10 transformed. Correlations between metabolites and microbial taxa at the ASV and Genus taxonomic levels were assessed using Spearman (stats package) correlation separately for each phenotype group. For metabolomics data, adjusted *Q* < 0.1 was considered to be statistically significant. Important correlations (Spearman correlation coefficient >0.25 and unadjusted *P*-value <0.005) were visualized as networks using network and ggnetwork packages.^56^ The correlation results were visualized as heat maps using the ComplexHeatmaps package.^57^ For data processing, visualization, and reporting of both microbiota and metabolome data, R packages tidyverse,^58^ ggsignif,^59^ broom,^60^ and knitr^61^ were used.

## Results

### Participant characteristics

In total, 877 women and men (body mass index (BMI): 25–40 kg/m^2^, aged 40–75 year) were fully screened for eligibility between May 2018 and November 2021.^6^ Tissue-specific IR was assessed during a 7-point oral glucose tolerance test (OGTT) by calculating the muscle insulin sensitivity index (MISI) and hepatic IR index (HIRI).^28,29^ Individuals were classified with predominant LIR or MIR using tertile cutoffs based on a previous study.^9,30^ After exclusion of 9 out of 242 randomized participants from analyses due to missing or low-quality fecal samples, we included data of 233 women and men with complete microbiota profiles (89 LIR and 144 MIR; 16S rRNA gene sequencing, V3-V4 region).

General characteristics were similar between LIR and MIR phenotypes (Table 1). The LIR group tended to have a lower percentage of women compared to the MIR group (LIR: 51.7% and MIR: 62.5%, *P* = 0.068). The LIR phenotype was characterized by a higher BMI and waist circumference (*P* = 0.024 and *P* = 0.030 respectively). Fasting plasma glucose (*P* = 0.003) and insulin (*P* = 0.047) were higher in LIR, while 2-hour glucose (*P* < 0.001) and insulin (*P* < 0.001) were higher in MIR. Concomitant with IR classification criteria, MISI and HIRI were higher in the LIR group (both (*P* < 0.001). The other OGTT-derived indices HOMA-IR (*P* = 0.019) and Disposition Index (*P* < 0.001) were also higher in LIR. There was a trend toward a higher Matsuda Index in MIR (*P* = 0.053). Relatively more people with LIR were classified as IFG, while more people with MIR were classified as IGT. In both groups, most people could be classified as normal glucose tolerant (NGT). There were relatively more people using anti-inflammatory medication in the MIR group (*P* = 0.023). Total energy intake was higher in the LIR group (*P* = 0.022), which may be related to a higher percentage of men (Table S1). The macronutrient breakdown of habitual dietary intake and physical activity were similar between groups. In both groups, most individuals had a normal self-reported stool consistency (BSS type 3 and 4) (Table S1).

**Table 1.** Characteristics of study participants stratified by tissue-specific IR phenotype.

	LIR phenotype	MIR phenotype	*P*-value
	n = 89	n = 144	
**General characteristics**			
Age, years	59.6 ± 7.3	60.6 ± 8.1	0.344
Women, n (%)	46 (51.7%)	90 (62.5%)	0.068
Systolic blood pressure, mmHg	127 ± 16	126 ± 14	0.754
Diastolic blood pressure, mmHg	80 ± 11	79 ± 10	0.600
**Anthropometrics and body composition**			
BMI, kg/m^2^	29.7 ± 3.6	29.5 ± 3.2	**0.024**
Waist circumference, cm	103.9 ± 10.8	101.0 ± 8.8	**0.030**
Waist-to-hip ratio	0.95 ± 0.09	0.93 ± 0.09	0.200
Body fat, %	36.3 ± 7.1	38.0 ± 7.7	0.104
VAT, L (LIR: *n* = 44, MIR: *n* = 47)	6.2 ± 2.2	5.4 ± 2.2	0.084
**Glucose homeostasis**			
Fasting glucose, mmol/L	5.7 [5.2, 6.0]	5.4 [5.1, 5.7]	**0.003**
2-hour glucose, mmol/L	5.9 [5.2, 7.0]	7.0 [5.9, 8.4]	**<0.001**
Fasting insulin, pmol/L	58.0 [45.2, 75.4]	52.5 [42.1, 69.5]	**0.047**
2-hour insulin, pmol/L	374.3 [229.2, 598.3]	511.1 [334.1, 823.0]	**<0.001**
HOMA-IR, AU	2.0 [1.6, 2.8]	1.8 [1.4, 2.5]	**0.019**
HOMA-β, AU	80.7 [61.2, 106.0]	78.4 [61.0, 100.4]	0.661
Disposition Index, AU	451.7 [260.8, 629.2]	303.4 [213.4, 471.0]	**<0.001**
Matsuda index, AU	9.4 [7.0, 13.2]	10.5 [7.9, 13.5]	0.053
MISI, AU	0.126 [0.106, 0.180]	0.070 [0.0478, 0.092]	**<0.001**
HIRI, AU	626 [500, 780]	344 [272, 442]	**<0.001**
**Glucose status, n (%)**			**<0.001**
NGT	63 (70.8%)	93 (64.6%)	
IFG	11 (12.4%)	2 (1.4%)	
IGT	5 (5.6%)	32 (22.2%)	
IGT+IFG	6 (6.7%)	6 (4.2%)	
T2DM	4 (4.5%)	11 (7.6%)	
**Medication use, n (%)**			
Antidepressants	7 (7.9%)	8 (5.6%)	0.331
Antihypertensives	12 (13.5%)	30 (20.8%)	0.106
Anti-inflammatory	4 (4.5%)	19 (13.2%)	**0.023**
Statins	5 (5.6%)	11 (7.6%)	0.379
Other	27 (30.3%)	50 (34.7%)	0.293

Differences between IR phenotypes were assessed using independent T-test for normally distributed numerical data (mean ± SD), Mann-Whitney test for non-normally distributed numerical data (median [25th percentile, 75th percentile], and using Fisher’s exact test for categorial data (n [%]).*P < 0.05 for difference between LIR and MIR phenotypes (bold). AU, arbitrary unit; BMI, body mass index; VAT, visceral adipose tissue; HOMA-IR, homeostatic model assessment of insulin resistance; HOMA- β, homeostatic model assessment of β-cell function; MISI, muscle insulin sensitivity index; HIRI, hepatic insulin resistance index; NGT, normal glucose tolerant; IGT, impaired glucose tolerance; IFG, impaired fasting glucose; T2D, type 2 diabetes.

### Microbial community differences between LIR and MIR phenotypes

**Figure 1.**
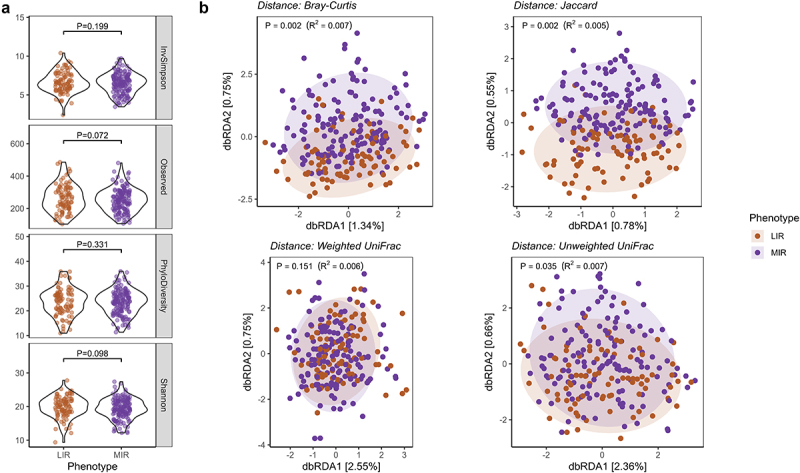
Microbial diversity and microbial community structure in people with LIR and MIR. (a) Box plots show alpha-diversity and richness as indicated by the inverse Simpson Index, number of observed taxa phylogenetic diversity, and Shannon Index. (b) Distance-based redundancy analysis (dbRDA) plots show beta-diversity as indicated by Bray-Curtis dissimilarity index, Jaccard index, weighted UniFrac and unweighted UniFrac. P-values for PERMANOVA with adjustment for age, sex and study center, total energy intake, fiber intake and Bristol stool scale for beta-diversity (b). LIR, liver insulin resistance; MIR, muscle insulin resistance. *P* < 0.05.

Microbial diversity and richness were similar between the LIR and MIR phenotypes, as indicated by the Inverse Simpson Index (*P* = 0.199), phylogenetic diversity (*P* = 0.331).There was a trend toward higher total observed taxa (*P* = 0.072) and Shannon Index (*P* = 0.098) in the LIR phenotype (Figure 1(a)). In addition, the LIR and MIR phenotypes were characterized by a distinct overall microbial community composition, as indicated by statistically significant PERMANOVA (*adonis2*) models with Bray-Curtis (*P* = 0.002, R^2^_phenotype_ = 0.007) and Jaccard (*P* = 0.002, R^2^_phenotype_ = 0.005) distances and Unweighted UniFrac (*P* = 0.035, R^2^_phenotype_ = 0.007) (Figure 1(b)). The model with weighted (*P* = 0.151, R^2^_phenotype_ = 0.006) distance was not different between the phenotypes. The covariates age and Bristol Stool Score significantly contributed to all beta diversity indices, while the contributions of study center, sex, and habitual daily energy and fiber intake were either less consistent or non-significant (Table S2).

### Differences in gut microbiota taxa between LIR and MIR phenotypes

**Figure 2.**
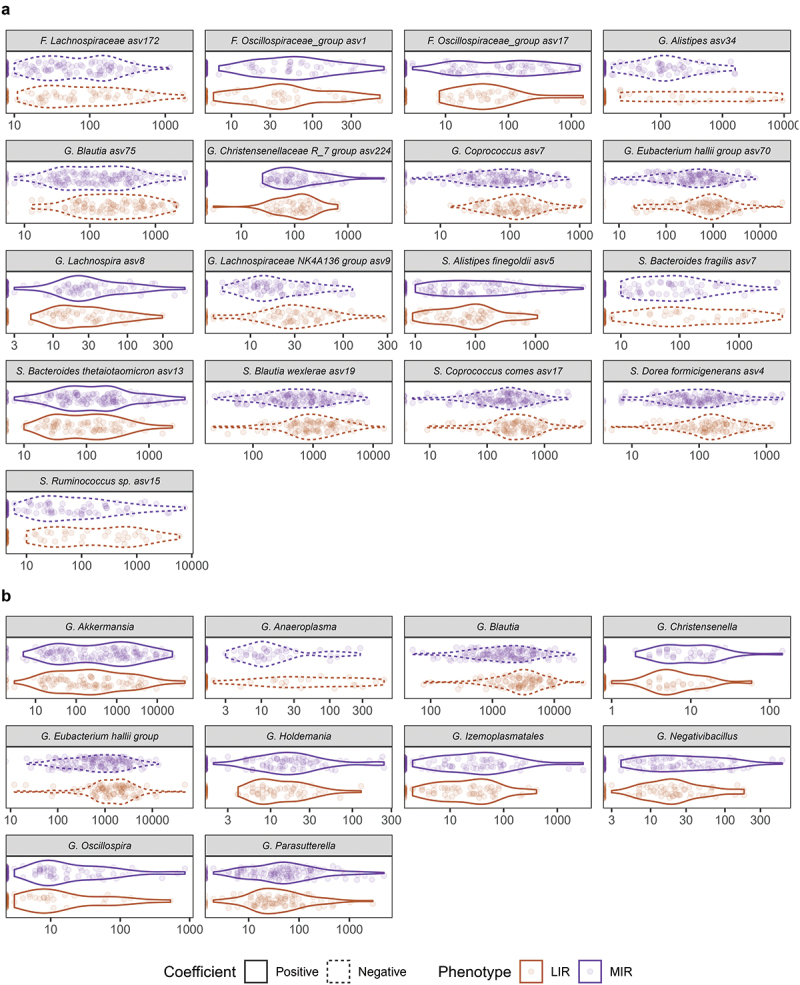
Differentially abundant gut microbial taxa between the LIR and MIR phenotypes. The trimmed mean of *M* values (TMM) normalized abundances of significantly different bacterial taxa at (a) ASV level and (b) genus level. Differences between liver IR and muscle IR were tested using zero-inflated binominal mixed models with adjustment for age, total energy intake, dietary fiber intake and Bristol stool scale (fixed variables), as well as sex and center (random variables). *P*-values were adjusted for multiple testing using Benjamini-Hochberg FDR. Adjusted *Q*-values < 0.1 were considered statistically different. Dotted lines indicate a negative coefficient, corresponding with higher abundance in LIR. ASV, amplicon sequence variant; LIR, liver insulin resistance; MIR, muscle insulin resistance.

In total there were 17 amplicon sequence variants (ASVs) with a differential abundance in LIR versus MIR phenotype, with 11 ASVs higher and 6 lower in the LIR compared to MIR phenotype (Figure 2(a)). These ASVs are closely linked to the differentially abundant genera in Figure 2(b) (Table S3). At genus level, we identified 10 bacterial taxa with a differential abundance between LIR and MIR, of which 3 genera with a higher relative abundance in the LIR phenotype, and 7 with a higher relative abundance in the MIR phenotype (Figure 2(a)). In LIR, the genera *Anaeroplasma, Blautia and Eubacterium Halli group* were higher compared to MIR. The MIR phenotype was characterized by a higher abundance of the genera *Akkermansia*, *Christensenella*, *Holdemania*, *Izemoplasmatales*, *Negativbacillus*, *Oscillospira*, and *Parasutterella*.

### Differences in functional indicators of microbial activity between LIR and MIR phenotypes

**Figure 3.**
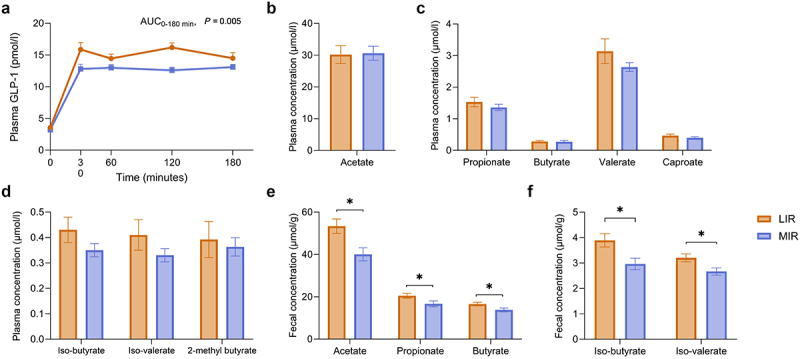
Plasma and fecal concentrations of gut-derived metabolites in people with LIR and MIR. (a) Plasma GLP-1 concentrations in fasting condition (*t* = 0) and postprandial after the ingestion of a HFMM (*n* = 160). (b-d). Plasma SCFA and BCFA concentrations (*n* = 82, MUMC+ center only). (e-f) Fecal SCFA and BCFA concentrations (*n* = 117, MUMC+ center only). Values are presented as mean ± SEM. Differences between muscle and liver IR were tested using linear mixed models with adjustment for age, sex, center, total energy intake, fiber intake and Bristol stool scale for plasma GLP-1 and age, sex, total energy intake, fiber intake and Bristol stool scale for plasma and fecal SCFAs and BCFAs. *P*-values were adjusted for multiple testing using Benjamini-Hochberg FDR. Statistical significance if *FDR adjusted *Q* < 0.05. BCFA, branch-chain fatty acid; GLP-1, glucagon-like peptide-1; HFMM, high-fat mixed-meal; LIR, liver insulin resistance; MIR, muscle insulin resistance; SCFA, short-chain fatty acid; SEM, standard error of the mean.

Next, we aimed to investigate whether the differences in overall microbial community structure and gut microbiota taxa between LIR and MIR phenotypes translated into differences in plasma and fecal gut metabolites between these phenotypes. The postprandial GLP-1 response after a liquid high-fat mixed-meal (HFMM, 350 g containing 2.8 MJ, 49 g [64 en%] fat, 48 g [29 en%] CHO, 12 g [7 en%] protein) was higher in the LIR than MIR phenotype (AUC_0-180 min_, *Q* = 0.005) (Figure 3(a)). No differences between LIR and MIR were found in fasting GLP-1 (*Q* = 0.526) and in the plasma SCFAs acetate (*Q* = 0.550), propionate (*Q* = 0.550), butyrate (*Q* = 0.550), valerate (*Q* = 0.489), caproate (*Q* = 0.550) and the plasma BCFAs iso-butyrate (*Q* = 0.489), iso-valerate (*p* = 0.550) and 2-methyl butyrate (*Q* = 0.550) (Figure 3(a–d)). However, fecal acetate (*Q* = 0.001), propionate (*Q* = 0.001) and butyrate (*Q* = 0.001), and fecal iso-butyrate (*Q* = 0.001) and iso-valerate (*Q* = 0.001) concentrations were higher in the LIR than MIR phenotype (Figure 3(e,f)).

### Metabolome profile and correlations with fecal bacteria

**Figure 4.**
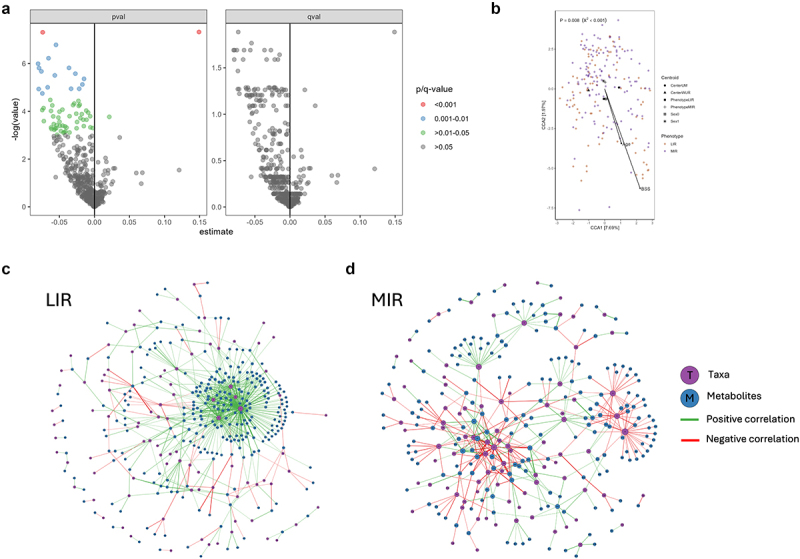
Plasma metabolome in LIR versus MIR and correlations with bacterial taxa on genus level. (a) Visual representation of *P*-values (left) and FDR-adjusted *Q*-values (right) of individual metabolites. Linear mixed-effect models included sex and study center as random variable and phenotype, age, Bristol stool score, habitual energy intake, and habitual fiber intake as fixed variables. Negative estimates indicate higher concentrations in LIR. *Q* < 0.1. (b) Canonical correspondence analysis (CCA) of the plasma metabolome profile between the LIR and MIR phenotypes, using the same parameters as constraining factors described in (a). Significance *P* < 0.05.(c,d) Network visualization of Spearman’s rank correlation analysis between metabolites and bacterial taxa at genus level in the LIR (c) and (d) MIR phenotypes. Only important correlations >0.25 with an unadjusted *P*-value < 0.005 are shown.

Additionally, we investigated whether tissue-specific IR is accompanied by specific plasma metabolome patterns. In total, 454 individual metabolites passed the quality check and were included for analysis. Before FDR-adjustment, 64 metabolites (indole SO_4_, ornithine, citrate, lipids and lipid derivates) appeared to have differential concentrations in LIR compared to MIR (*P* < 0.05), which disappeared after adjustment for multiple testing (*Q* < 0.1) (Figure 4(a),Table S4). However, using CCA analysis, we identified a distinct metabolite pattern in the LIR versus MIR phenotype (*P* = 0.008, Log_10_ transformed normalized count) (Figure 4(b)).

Next, we assessed correlations between the bacterial taxa and the plasma metabolome, plasma GLP-1 and SCFAs, BCFAs in plasma or feces, which is visualized as structural networks in Figure 4(c,d). There are distinct network hubs in the LIR versus MIR phenotypes. In LIR (Figure 4(c)), there are mainly positive network hubs related to the genera *Alistipes* (correlated with *n* = 150 metabolites), *Holdemania* (correlated with *n* = 90 metabolites), *Bilophila* (correlated with *n* = 63 metabolites), *Bacteroides* (correlated with *n* = 49 metabolites) and *Barnesiella* (correlated with *n* = 49 metabolites) (Table S5). The network hubs in MIR are less interconnected and are characterized by both positive and negative correlations (Figure 4(d)).

In addition, the LIR and MIR phenotypes showed distinct patterns in the types of correlations between microbial genera and either plasma or fecal metabolites (Figure S1). Only in the LIR phenotype, the genera *Alistipes*, *Bilophila* and *Holdemania* were positively and the genus *Desulfovibrio* negatively related to a range of lipid compounds (Figure S1A). In addition, there was a negative correlation between *Butyricicoccus* and p-cresol SO_4._ In MIR, only the genus *Oscilospirales 010* was negatively correlated to 3 lipid compounds. On the other hand, in contrast to LIR, the MIR phenotype was characterized by more prominent and merely positive correlations between microbial taxa and plasma and fecal SCFA and BCFA (Figure S1C-D). We did not find any correlations between plasma GLP-1 and microbial taxa in both the LIR and MIR phenotypes (data not shown). Overall, at genus level, all correlations were unique to either MIR or LIR and related to specific functional subsets of metabolites.

### Differential microbiota composition in LIR and MIR compared to a ‘No IR’ phenotype

We next determined whether LIR and MIR had a distinct microbiota composition compared to a reference cohort consisting of individuals without LIR or MIR (‘No IR’ phenotype, Methods section). The LIR and MIR phenotypes had a higher age, fasting plasma glucose and insulin compared to the ‘No IR’ group (Table S6). Furthermore, individuals with LIR had a higher waist circumference and a higher HIRI value compared to those with ‘No IR’. The 2-hour plasma glucose concentration and MISI was higher only in people with MIR compared to people with ‘No IR’.

**Figure 5.**
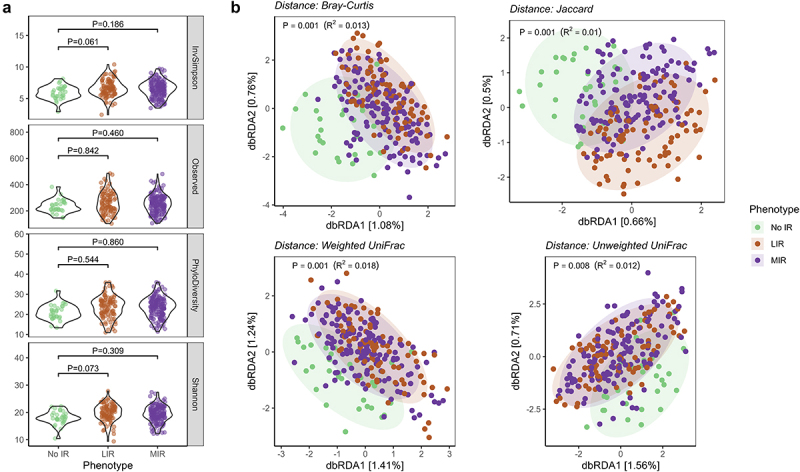
Microbial diversity and microbial community structure in people with LIR and MIR compared to ‘No IR’. (a) Box plots show alpha-diversity and richness as indicated by the inverse Simpson index, number of observed taxa, phylogenetic diversity and Shannon index. (b) Distance-based redundancy analysis (dbRDA) plots show beta-diversity as indicated by the Bray-Curtis dissimilarity, Jaccard index and weighted and unweighted UniFrac. *P*-values for alpha diversity and richness (a) are for linear mixed models, and PERMANOVA (b) with adjustment for age, sex, study center. LIR, liver insulin resistance; MIR, muscle insulin resistance. *P* < 0.05.

Microbial diversity, reflected by the Inverse Simpson Index (LIR, *P*= 0.061; MIR, *P* = 0.186), number of observed taxa (LIR, *P* = 0.842; MIR, *P* = 0.461), phylogenetic diversity (LIR, *P* = 0.544; MIR, *P* = 0.860) and Shannon Index (LIR, *P* = 0.073; MIR, *P* = 0.309), were no different between LIR, MIR, and No IR phenotypes (Figure 5(a)). However, we identified distinct microbial community structures between the 3 metabolic phenotypes for Bray-Curtis Dissimilarity Index (*P* = 0.001, R^2^_phenotype_ = 0.013), Jaccard Index (*P* = 0.001, R^2^_phenotype_ = 0.010), Weighted UniFrac (*P* = 0.001, R^2^_phenotype_ = 0.018), and Unweighted UniFrac (*P* = 0.008, R^2^_phenotype_ = 0.012) (Figure 5(b)).

## Discussion

In this cross-sectional study, we investigated whether people with LIR compared to MIR have distinct gut microbiota features. Both gut microbiota composition and concentrations of functional markers of gut microbiota activity were determined in deeply-phenotyped individuals with overweight or obesity with predominant LIR or MIR. We demonstrated that these phenotypes are characterized by a distinct gut microbial composition and gut-related metabolite profile. Interestingly, the overall microbial community structure of LIR and MIR differed from a reference cohort without IR as well. Individuals with LIR showed enrichment of individual beneficial, potent SCFA producing bacterial taxa compared to individuals with MIR. In line, fecal SCFA and BCFA concentrations and the postprandial plasma GLP-1 response, linked to gut microbiota activity as functional markers, were also higher in individuals with LIR compared to MIR. Finally, we found differences in microbiota-metabolites correlational networks in individuals with LIR versus MIR.

Our present study showed differences in overall microbial community structure between LIR and MIR phenotypes, which was independent of important confounders such as age, sex, geographic location, Bristol Stool Score and habitual dietary intake.^62^ Still, in this model the explained variance for tissue-specific IR phenotype was relatively small, indicating the high individuality of the gut microbiota. However, differences in individual microbes did point toward MIR and LIR-specific microbial phenotypes. We found differences in several microbial taxa abundances between LIR and MIR. More specifically, *Eubacterium hallii* group, *Blautia* and *Anaeroplasma* genera were more abundant in the LIR phenotype. These taxa, which reflect approximately 5% of the total microbiota community at genus level in LIR, have been established as SCFA producers and have been linked to beneficial health effects, including improved insulin sensitivity in mice^63^ and lower visceral fat mass in adults.^64,65^ Interestingly, the abundance of the genus *Akkermansia*, which is well known for having a positive effect on human health,^66^ was higher in MIR. Overall, we could not translate this finding into functional markers of gut microbiota activity in this cohort.

The higher functional potential of compositional features in individuals with the LIR phenotype may be reflected by her concentrations of fecal SCFAs, BCFAs and postprandial plasma GLP-1 in individuals with LIR compared to MIR. Additionally, the higher postprandial plasma GLP-1 response may point toward higher SCFA-stimulated GLP-1 secretion by intestinal L-cells in LIR compared to MIR.^67^ Although the combination of microbial composition with abundant SCFA-producing taxa as well as increased fecal SCFA concentrations indicate a higher SCFA production in LIR, circulating concentrations were similar between LIR and MIR. Possibly, SCFAs may be taken up, metabolized or excreted faster in either one of the phenotypes, making it difficult to assess the true total production of SCFAs in an *in vivo* setting. Given the role of GLP-1 in regulating glucose metabolism,^68^ our data support the idea that the gut microbiota-related features in people with LIR are more favorable compared to MIR.

Importantly, these findings highlight potential difference contributors to IR in the liver versus muscle. Whereas LIR is characterized by a more beneficial gut microbial profile, we previously reported a greater disturbance of the blood lipid profile and higher visceral fat content in this phenotype.^69,70^ The phenotype-specific microbiota – plasma metabolites correlational patterns may further confirm the distinct interplay between the gut microbiota and metabolic effects in tissue-specific IR. Here, the genera *Alistipes*, *Bilophila* and *Holdemania* were positively and the genus *Desulfovibrio* negatively related to a range of lipid compounds, while we did not detect a similar pattern in the MIR phenotype. This would be in line with previously confirmed LIR-specific abnormalities in the plasma lipid profile.^71^ This may suggest that people with MIR could benefit more from gut microbiota-targeted interventions to improve cardiometabolic health, while LIR may benefit more from other types of interventions. However, these underlying metabolic mechanisms of tissue-specific IR linked to the gut microbiota should be investigated further.

Lastly, we also found that the overall microbial community structure in people with LIR and MIR were different from a reference population without LIR or MIR. These phenotype-dependent microbial community differences are in line with previous studies, in which significant gut microbiota alterations were found in individuals with obesity, IR and T2D compared to healthy controls.^72,73^ It has been reported that the gut microbial profile of people with especially impaired glucose tolerance (IGT), who are characterized by worse peripheral (muscle) insulin sensitivity, have a more deviating microbial profile compared to individuals with normoglycemia and impaired fasting glucose, pointing toward a more dysbiotic state associated with MIR.^20^ It remains to be determined to what extent fasting and postprandial gut metabolite profiles in LIR and MIR differ from other phenotypes such as individuals without IR or those with T2D.

A major strength of the present study is the extensive metabolic phenotyping in individuals with tissue-specific IR, as well as the assessment of several parameters related to gut microbiota activity, in addition to gut microbiota composition. Nevertheless, our study also has several limitations. First, the sample size (*n* = 233) is relatively small compared to other observational studies. Importantly, the smaller reference cohort without tissue-specific IR was not part of the original study design and was included post-hoc. Although the study participants were matched to clinical parameters and date of collection of the main cohort, we could have still introduced some unknown bias and variation to the analyses. Secondly, bacterial metabolites including fecal and plasma SCFA and BCFA were measured in a subgroup of the study population with the LIR and MIR phenotypes, which may have impacted the study findings. Hence, the current findings require replication in larger, more diverse cohorts. Future work related to metabotyping and concomitant gut microbiota profiling may include metagenomic sequencing to be able to improve higher taxonomic resolution and to deepen the mechanistic understanding of the role of the gut microbiota in relation to the heterogeneity in tissue-specific IR and metabolic traits.^74^ Another limitation of this study is the use of OGTT-derived measures for assessment of tissue-specific IR. The estimated MISI and HIRI have been validated against the gold-standard clamp^28^ and have been previously used in intervention studies and cohorts. Contrary to standardized clamp-derived insulin sensitivity measures, MISI and HIRI may to some extent be determined by other biological processes such as the rate of glucose absorption as well as the incretin response. Furthermore, OGTT-derived indices are known for their modest reproducibility.^75^ Nevertheless, previous data show robust and reproducible distinct metabolome, lipidome, abdominal adipose tissue transcriptome and systemic inflammatory profiles in MIR and LIR defined with one OGTT.^7,9,71^

## Conclusion

The present study demonstrates for the first time that LIR and MIR phenotypes are characterized by differential gut microbial composition and gut metabolite profile in humans with overweight or obesity. We observed higher abundance of bacteria that have previously been linked to SCFA production and good metabolic health, as well as higher gut-derived metabolites (GLP-1, SCFAs, BCFAs) in LIR compared to MIR. This points toward an overall more favorable microbial profile in LIR compared to MIR, which may indicate that people with MIR may be more susceptible to gain health benefits specifically through gut microbiota modulation. Together, the present findings support a role of the gut microbiota as modifiable factor underlying tissue dysmetabolism and tissue-specific IR in individuals at increased risk for T2D. Future studies are needed to elucidate whether the microbiota composition in people with MIR, LIR and other cardiometabolic phenotypes may mediate tissue-specific improvements in insulin sensitivity after lifestyle or pharmacological intervention.

## Supplementary Material

Supplemental_information_v2.docx

## Data Availability

The published article and supplemental information contain the clinical data used to generate the figures in the paper. Data generated by 16S rRNA sequencing and corresponding deidentified participant metadata are deposited in the National Center for Biotechnology Information Sequence Read Archive (NCBI SRA) under accession code: PRJNA1011518. The complete microbiota analysis pipeline is published at https://github.com/AlexanderUm/PERSON_microbiome_and_phenotype. Any other information required to reanalyze the data reported in this paper is available from the lead contact upon reasonable request.
