# Supplementary material for: Distinct gut microbiota and metabolome features of tissue-specific insulin resistance in overweight and obesity
Source: Gut Microbes. 2025 May 7;17(1):2501185. doi: 10.1080/19490976.2025.2501185 (PMC12064058; doi:10.1080/19490976.2025.2501185)
Supplement: Supplemental_information_v2.docx [file KGMI_A_2501185_SM1928.docx]

# Supplementary information

## Table S1. Self-reported habitual dietary intake, physical activity and stool consistency stratified per tissue-specific IR phenotype. Related to Table 1.

|  | **LIR phenotype** | **MIR phenotype** | ***P*-value** |
| --- | --- | --- | --- |
|  |  | |  |
| **Habitual diet** | n = 77 | n = 125 |  |
| Total energy intake, MJ | 9.6 [7.7, 11.4] | 8.6 [7.4, 10.3] | **0.022** |
| Habitual fat intake, en% | 37.6 ± 6.5 | 37.7 ± 5.1 | 0.927 |
| Saturated fat | 14.0 ± 3.1 | 13.7 ± 2.5 | 0.489 |
| Monounsaturated fat | 13.5 ± 2.5 | 13.5 ± 2.5 | 0.958 |
| Polyunsaturated fat | 7.0 ± 1.7 | 7.3 ± 1.8 | 0.217 |
| Habitual protein intake, en% | 15.6 ± 2.0 | 15.8 ± 2.2 | 0.902 |
| Animal protein, % of total | 59.9 ± 8.7 | 58.6 ± 8.9 | 0.321 |
| Plant-based protein, % of total | 40.1 ± 8.7 | 41.4 ± 8.9 | 0.324 |
| Habitual CHO intake, en% | 41.1 ± 6.2 | 41.8 ± 5.4 | 0.398 |
| Mono- and disaccharides | 18.8 ± 5.8 | 19.1 ± 4.9 | 0.614 |
| Polysaccharides | 22.3 ± 5.0 | 22.6 ± 4.6 | 0.641 |
| Habitual fiber intake, g/MJ | 2.5 ± 0.6 | 2.6 ± 0.6 | 0.724 |
| Habitual alcohol consumption, g | 7.4 [1.5, 15.8] | 5.5 [2.5, 11.1] | 0.509 |
| **Habitual physical activity** | n = 87 | n = 142 |  |
| Baecke score | 8.5 [7.4, 9.4] | 8.3 [7.4, 9.1] | 0.498 |
| **Intestinal health** | n = 88 | n = 143 |  |
| Bristol stool score | 4 [3, 5] | 4 [3, 5] | 0.103 |
| BSS type 1, n (%) | 0 (0.0%) | 2 (1.4%) |  |
| BSS type 2, n (%) | 8 (9.1%) | 12 (8.4%) |  |
| BSS type 3, n (%) | 21 (23.9%) | 48 (33.6%) |  |
| BSS type 4, n (%) | 22 (25.0%) | 44 (30.8%) |  |
| BSS type 5, n (%) | 18 (20.5%) | 23 (16.1%) |  |
| BSS type 6, n (%) | 18 (20.5%) | 14 (9.8%) |  |
| BSS type 7, n (%) | 1 (1.1%) | 0 (0.0%) |  |

Differences between IR phenotypes were assessed using independent T-test for normally distributed numerical data (mean ± SD), Mann-Whitney test for non-normally distributed numerical data (median [25th percentile, 75th percentile], and using Fisher’s exact test for categorial data (n [%]).**P* < 0.05 for difference between LIR and MIR phenotypes. En%, energy percentage of total energy intake; MJ, megajoule; CHO carbohydrates; BSS, Bristol Stool Scale.

## Table S2. Summary statistics for Permutational Multivariate Analysis of Variance Using Distance Matrices (adonis2). Related to Figure 1.

|  | **Term** | **Sum of squares** | **R2** | **F** | ***P-*value** |
| --- | --- | --- | --- | --- | --- |
| Bray-Curtis | Age | 0.364 | 0.007 | 1.59 | 0.005 |
|  | BSS | 0.476 | 0.009 | 2.08 | 0.001 |
|  | Energy Intake | 0.204 | 0.004 | 0.89 | 0.786 |
|  | Fiber Intake | 0.256 | 0.005 | 1.12 | 0.151 |
|  | Study Center | 0.362 | 0.007 | 1.58 | 0.006 |
|  | Sex | 0.347 | 0.006 | 1.52 | 0.008 |
|  | Phenotype | 0.361 | 0.007 | 1.58 | 0.002 |
|  | Residual | 51.526 | 0.956 | NA | NA |
|  | Total | 53.897 | 1.000 | NA | NA |
|  |  |  |  |  |  |
|  | **Term** | **Sum of squares** | **R2** | **F** | ***P-*value** |
| Jaccard | Age | 0.498 | 0.005 | 1.26 | 0.002 |
|  | BSS | 0.538 | 0.006 | 1.36 | 0.001 |
|  | Energy Intake | 0.385 | 0.004 | 0.97 | 0.680 |
|  | Fiber Intake | 0.419 | 0.005 | 1.06 | 0.132 |
|  | Study Center | 0.478 | 0.005 | 1.21 | 0.004 |
|  | Sex | 0.471 | 0.005 | 1.19 | 0.003 |
|  | Phenotype | 0.490 | 0.005 | 1.24 | 0.002 |
|  | Residual | 88.944 | 0.964 | NA | NA |
|  | Total | 92.222 | 1.000 | NA | NA |
|  |  |  |  |  |  |
|  | **Term** | **Sum of squares** | **R2** | **F** | ***P-*value** |
| Unweighted UniFrac | Age | 0.343 | 0.011 | 2.60 | 0.001 |
|  | BSS | 0.401 | 0.013 | 3.05 | 0.001 |
|  | Energy Intake | 0.138 | 0.004 | 1.05 | 0.337 |
|  | Fiber Intake | 0.137 | 0.004 | 1.04 | 0.343 |
|  | Study Center | 0.218 | 0.007 | 1.66 | 0.027 |
|  | Sex | 0.185 | 0.006 | 1.40 | 0.059 |
|  | Phenotype | 0.206 | 0.007 | 1.56 | 0.035 |
|  | Residual | 29.647 | 0.948 | NA | NA |
|  | Total | 31.275 | 1.000 | NA | NA |
|  |  |  |  |  |  |
|  | **Term** | **Sum of squares** | **R2** | **F** | ***P-*value** |
| Weighted UniFrac | Age | 0.090 | 0.009 | 2.24 | 0.009 |
|  | BSS | 0.165 | 0.017 | 4.12 | 0.001 |
|  | Energy Intake | 0.026 | 0.003 | 0.65 | 0.868 |
|  | Fiber Intake | 0.038 | 0.004 | 0.95 | 0.500 |
|  | Study Center | 0.065 | 0.007 | 1.62 | 0.051 |
|  | Sex | 0.058 | 0.006 | 1.45 | 0.100 |
|  | Phenotype | 0.053 | 0.006 | 1.32 | 0.151 |
|  | Residual | 8.993 | 0.948 | NA | NA |
|  | Total | 9.486 | 1.000 | NA | NA |

Beta diversity as calculated by PERMANOVA (adonis2) using the model *Distance Matrix ~ Age + BSS + Energy Intake + Fiber Intake + Study Center + Sex + Phenotype* with 999 unconstrained permutations. BSS, Bristol Stool Score; LIR, liver insulin resistance; MIR, muscle insulin resistance. P < 0.05.

## Table S3. Genera and ASVs significantly different in abundance between the LIR and MIR phenotypes. Related to Figure 2.

|  | **LIR phenotype (n = 89)** | | **MIR phenotype (n = 144)** | |  | | | |
| --- | --- | --- | --- | --- | --- | --- | --- | --- |
|  | **Median (%)** | **IQR (%)** | **Median (%)** | **IQR (%)** | **Coeffi- cient** | **St. error** | ***P-***  **value** | ***Q-***  **value** |
| **Genus** | | | | | | | | |
| ***LIR ↑*** | | | | | | | | |
| *G. Anaeroplasma* | 0.000 | 0.000 | 0.000 | 0.007 | -1.151 | 0.307 | 0.000 | 0.010 |
| *G. Eubacterium hallii group* | 2.394 | 3.469 | 1.541 | 2.909 | -0.447 | 0.142 | 0.002 | 0.041 |
| *G. Blautia* | 4.527 | 4.838 | 3.345 | 4.318 | -0.310 | 0.111 | 0.005 | 0.079 |
| ***MIR ↑*** | | | | | | | | |
| *G. Holdemania* | 0.007 | 0.018 | 0.007 | 0.029 | 0.552 | 0.181 | 0.002 | 0.045 |
| *G. Akkermansia* | 0.255 | 1.676 | 0.480 | 3.199 | 0.658 | 0.244 | 0.007 | 0.091 |
| *G. Negativibacillus* | 0.010 | 0.038 | 0.015 | 0.052 | 0.682 | 0.175 | 0.000 | 0.009 |
| *G. Parasutterella* | 0.044 | 0.108 | 0.057 | 0.148 | 0.737 | 0.194 | 0.000 | 0.009 |
| *G. Christensenella* | 0.000 | 0.003 | 0.000 | 0.002 | 0.842 | 0.240 | 0.000 | 0.021 |
| *G. Izemoplasmatales* | 0.006 | 0.039 | 0.000 | 0.040 | 1.042 | 0.366 | 0.004 | 0.070 |
| *G. Oscillospira* | 0.000 | 0.003 | 0.000 | 0.013 | 1.119 | 0.391 | 0.004 | 0.070 |
| **ASV** | | | | | | | | |
| ***LIR ↑*** | | | | | | | | |
| *G. Alistipes asv34* | 0.000 | 0.000 | 0.000 | 0.158 | -1.695 | 0.400 | 0.000 | 0.003 |
| *S. Bacteroides fragilis asv7* | 0.000 | 0.020 | 0.000 | 0.051 | -1.308 | 0.395 | 0.001 | 0.034 |
| *S. Ruminococcus sp. asv15* | 0.000 | 0.064 | 0.000 | 0.088 | -1.148 | 0.395 | 0.004 | 0.091 |
| *G. Eubacterium hallii group asv70* | 1.149 | 2.331 | 0.771 | 1.430 | -0.676 | 0.152 | 0.000 | 0.003 |
| *F. Lachnospiraceae asv172* | 0.027 | 0.135 | 0.039 | 0.183 | -0.628 | 0.167 | 0.000 | 0.013 |
| *G. Blautia asv75* | 0.324 | 0.858 | 0.223 | 0.436 | -0.612 | 0.140 | 0.000 | 0.003 |
| *G. Lachnospiraceae NK4A136 group asv9* | 0.023 | 0.056 | 0.006 | 0.030 | -0.507 | 0.125 | 0.000 | 0.005 |
| *S. Blautia wexlerae asv19* | 1.549 | 2.346 | 1.050 | 1.976 | -0.467 | 0.119 | 0.000 | 0.007 |
| *S. Coprococcus comes asv17* | 0.593 | 0.529 | 0.407 | 0.469 | -0.345 | 0.117 | 0.003 | 0.085 |
| *G. Coprococcus asv7* | 0.170 | 0.189 | 0.127 | 0.167 | -0.336 | 0.102 | 0.001 | 0.035 |
| *S. Dorea formicigenerans asv4* | 0.242 | 0.222 | 0.170 | 0.273 | -0.309 | 0.106 | 0.004 | 0.091 |
| ***MIR ↑*** | | | | | | | | |
| *G. Christensenellaceae R_7*  *group asv224* | 0.043 | 0.173 | 0.000 | 0.207 | 0.532 | 0.166 | 0.001 | 0.047 |
| *S. Bacteroides thetaiotaomicron asv13* | 0.158 | 0.290 | 0.218 | 0.428 | 0.551 | 0.161 | 0.001 | 0.026 |
| *G. Lachnospira asv8* | 0.000 | 0.027 | 0.000 | 0.044 | 0.599 | 0.194 | 0.002 | 0.063 |
| *F. Oscillospiraceae_group asv1* | 0.000 | 0.032 | 0.000 | 0.021 | 0.752 | 0.250 | 0.003 | 0.074 |
| *F. Oscillospiraceae_group*  *asv17* | 0.000 | 0.025 | 0.000 | 0.078 | 0.978 | 0.313 | 0.002 | 0.058 |
| *S. Alistipes finegoldii asv5* | 0.038 | 0.146 | 0.025 | 0.206 | 1.108 | 0.259 | 0.000 | 0.003 |

Relative abundances of significantly different bacterial taxa at ASV level and genus level. Differences between liver IR and muscle IR were tested using zero-inflated binominal mixed models with adjustment for age, total energy intake, dietary fiber intake and Bristol Stool Score (fixed variables), as well as sex and center (random variables). P-values were adjusted for multiple testing using Benjamini- Hochberg FDR. Adjusted Q-values < 0.1 were considered statistically different. Negative coefficients indicate a higher abundance in LIR. ASV, amplicon sequence variant; LIR, liver insulin resistance; MIR, muscle insulin resistance.

## Table S4. Plasma metabolite concentrations stratified per LIR and MIR tissue-specific IR phenotype. Related to Figure 4.

|  | **LIR phenotype (n=83)** | | **MIR phenotype (n=124)** | |  |  |  |  |
| --- | --- | --- | --- | --- | --- | --- | --- | --- |
| **Metabolite** | **Median (µM)** | **IQR (µM)** | **Median (µM)** | **IQR (µM)** | **Esti- mate** | **Std. Error** | **P-**  **value** | **Q-**  **value** |
| Indole SO4 | 4.1 | 4.0 | 5.1 | 4.0 | 0.149 | 0.043 | 0.001 | 0.152 |
| DG_18_1_18_2 | 11.1 | 5.6 | 9.2 | 4.2 | -0.074 | 0.021 | 0.001 | 0.152 |
| DG_16_0_18_2 | 2.1 | 1.4 | 1.8 | 1.2 | -0.055 | 0.017 | 0.001 | 0.171 |
| DG_18_2_18_2 | 4.6 | 2.4 | 3.8 | 2.1 | -0.065 | 0.021 | 0.002 | 0.184 |
| TG_18_1_36_3 | 153.0 | 91.5 | 125.0 | 75.4 | -0.080 | 0.026 | 0.002 | 0.184 |
| Ornithine | 65.4 | 16.5 | 61.8 | 17.3 | -0.036 | 0.012 | 0.003 | 0.184 |
| TG_18_2_36_2 | 80.7 | 50.3 | 66.9 | 39.4 | -0.079 | 0.026 | 0.003 | 0.184 |
| TG_18_1_36_2 | 212.0 | 143.0 | 175.5 | 89.5 | -0.076 | 0.026 | 0.003 | 0.184 |
| TG_20_1_34_0 | 0.5 | 0.4 | 0.4 | 0.2 | -0.027 | 0.009 | 0.004 | 0.184 |
| DG_18_1_18_1 | 6.0 | 3.4 | 5.4 | 1.9 | -0.057 | 0.019 | 0.004 | 0.184 |
| Cer_d16_1_24_0 | 0.4 | 0.1 | 0.3 | 0.1 | -0.015 | 0.005 | 0.005 | 0.194 |
| TG_17_2_38_6 | 0.3 | 0.2 | 0.3 | 0.2 | -0.017 | 0.006 | 0.006 | 0.204 |
| TG_18_2_36_1 | 25.9 | 13.8 | 21.9 | 11.2 | -0.067 | 0.024 | 0.007 | 0.204 |
| Cer_d18_1_24_0 | 3.3 | 1.1 | 3.2 | 1.1 | -0.033 | 0.012 | 0.007 | 0.204 |
| TG_17_2_36_3 | 0.5 | 0.3 | 0.4 | 0.2 | -0.022 | 0.008 | 0.007 | 0.204 |
| TG_18_2_36_3 | 48.6 | 26.3 | 40.1 | 24.7 | -0.079 | 0.029 | 0.007 | 0.204 |
| TG_18_1_36_4 | 47.8 | 27.2 | 39.4 | 24.2 | -0.074 | 0.028 | 0.009 | 0.231 |
| TG_18_0_36_3 | 22.5 | 12.7 | 18.6 | 10.5 | -0.063 | 0.025 | 0.011 | 0.276 |
| TG_17_1_36_5 | 0.4 | 0.3 | 0.3 | 0.2 | -0.023 | 0.009 | 0.012 | 0.276 |
| PC_aa_C34_2 | 365.0 | 82.5 | 345.5 | 102.3 | -0.029 | 0.011 | 0.013 | 0.276 |
| TG_17_2_36_2 | 0.5 | 0.3 | 0.5 | 0.3 | -0.021 | 0.008 | 0.014 | 0.276 |
| TG_20_0_32_3 | 0.9 | 0.5 | 0.8 | 0.5 | -0.030 | 0.012 | 0.014 | 0.276 |
| TG_18_0_36_4 | 7.9 | 4.3 | 6.7 | 4.3 | -0.060 | 0.024 | 0.014 | 0.276 |
| TG_20_1_34_3 | 0.5 | 0.3 | 0.4 | 0.2 | -0.022 | 0.009 | 0.015 | 0.276 |
| TG_18_2_36_4 | 17.7 | 10.6 | 14.3 | 9.0 | -0.073 | 0.030 | 0.016 | 0.281 |
| TG_18_2_36_0 | 2.9 | 1.8 | 2.5 | 1.3 | -0.046 | 0.019 | 0.017 | 0.281 |
| SM_C16_1 | 15.2 | 4.0 | 15.0 | 6.4 | -0.074 | 0.031 | 0.017 | 0.281 |
| Cer_d18_2_22_0 | 0.4 | 0.1 | 0.4 | 0.1 | -0.012 | 0.005 | 0.018 | 0.281 |
| TG_18_1_36_1 | 55.8 | 39.1 | 50.2 | 25.9 | -0.062 | 0.026 | 0.018 | 0.281 |
| DG_16_0_18_1 | 4.3 | 2.8 | 3.8 | 2.4 | -0.050 | 0.021 | 0.019 | 0.281 |
| Cer_d16_1_20_0 | 0.1 | 0.1 | 0.1 | 0.1 | -0.009 | 0.004 | 0.019 | 0.282 |
| TG_17_2_36_4 | 0.8 | 0.5 | 0.8 | 0.4 | -0.026 | 0.011 | 0.020 | 0.283 |
| Cer_d18_2_24_0 | 0.8 | 0.3 | 0.7 | 0.3 | -0.018 | 0.008 | 0.021 | 0.283 |
| C2 | 7.2 | 2.7 | 6.7 | 2.7 | -0.036 | 0.015 | 0.022 | 0.283 |
| Cer_d18_1_20_0_ | 0.2 | 0.1 | 0.2 | 0.1 | -0.006 | 0.003 | 0.023 | 0.283 |
| TG_18_0_36_2_ | 21.9 | 16.2 | 19.6 | 9.7 | -0.059 | 0.026 | 0.023 | 0.283 |
| PC_ae_C44_5 | 1.2 | 0.3 | 1.3 | 0.4 | 0.021 | 0.009 | 0.023 | 0.283 |
| TG_18_2_35_1_ | 5.4 | 2.9 | 5.0 | 3.2 | -0.051 | 0.023 | 0.024 | 0.283 |
| PC_aa_C32_3 | 0.5 | 0.2 | 0.5 | 0.3 | -0.020 | 0.009 | 0.024 | 0.283 |

| Cer_d16_1_22_0_ | 0.6 | 0.2 | 0.5 | 0.2 | -0.016 | 0.007 | 0.026 | 0.288 |
| --- | --- | --- | --- | --- | --- | --- | --- | --- |
| Cer_d16_1_23_0_ | 0.3 | 0.1 | 0.2 | 0.1 | -0.011 | 0.005 | 0.026 | 0.288 |
| SM_C18_1 | 9.5 | 3.6 | 9.8 | 5.0 | -0.072 | 0.033 | 0.028 | 0.295 |
| TG_17_0_36_3_ | 5.1 | 3.2 | 4.9 | 2.9 | -0.050 | 0.023 | 0.028 | 0.295 |
| TG_20_0_32_4_ | 0.7 | 0.4 | 0.6 | 0.3 | -0.025 | 0.011 | 0.029 | 0.295 |
| TG_18_2_34_1_ | 302.0 | 180.0 | 255.5 | 147.8 | -0.058 | 0.027 | 0.031 | 0.300 |
| TG_18_2_33_1_ | 7.8 | 4.8 | 7.3 | 4.6 | -0.054 | 0.025 | 0.033 | 0.300 |
| TG_18_2_35_2_ | 3.5 | 1.9 | 3.2 | 2.0 | -0.045 | 0.021 | 0.033 | 0.300 |
| TG_18_1_33_2_ | 6.4 | 3.7 | 5.9 | 3.5 | -0.049 | 0.023 | 0.034 | 0.300 |
| Cer_d18_1_22_0_ | 1.1 | 0.3 | 1.1 | 0.4 | -0.015 | 0.007 | 0.034 | 0.300 |
| TG_17_1_36_3_ | 2.9 | 1.6 | 2.8 | 1.8 | -0.042 | 0.020 | 0.034 | 0.300 |
| TG_20_2_36_5_ | 0.1 | 0.1 | 0.1 | 0.1 | -0.006 | 0.003 | 0.036 | 0.300 |
| TG_18_2_34_2_ | 148.0 | 89.9 | 123.0 | 76.0 | -0.062 | 0.029 | 0.036 | 0.300 |
| TG_20_1_34_2_ | 2.2 | 1.4 | 1.9 | 1.1 | -0.040 | 0.019 | 0.036 | 0.300 |
| HexCer_d18_2_18_ 0 | 0.0 | 0.0 | 0.0 | 0.0 | -0.002 | 0.001 | 0.037 | 0.300 |
| TG_18_1_34_2_ | 404.0 | 224.0 | 351.5 | 205.5 | -0.055 | 0.026 | 0.037 | 0.300 |
| Citrate | 35.1 | 11.0 | 33.3 | 9.6 | -0.025 | 0.012 | 0.037 | 0.300 |
| TG_16_0_36_3_ | 335.0 | 207.0 | 285.0 | 162.3 | -0.056 | 0.027 | 0.038 | 0.300 |
| TG_16_0_36_4_ | 94.6 | 56.7 | 76.0 | 49.5 | -0.061 | 0.029 | 0.038 | 0.300 |
| TG_18_3_36_2_ | 13.2 | 8.2 | 10.9 | 7.3 | -0.057 | 0.028 | 0.040 | 0.305 |
| TG_18_1_35_2_ | 8.7 | 5.1 | 8.4 | 4.6 | -0.048 | 0.023 | 0.042 | 0.315 |
| TG_18_2_33_2_ | 2.3 | 1.3 | 2.2 | 1.5 | -0.041 | 0.020 | 0.042 | 0.315 |
| TG_20_1_34_1_ | 3.6 | 2.5 | 3.2 | 1.6 | -0.043 | 0.021 | 0.044 | 0.318 |
| TG_17_0_36_4_ | 1.6 | 0.8 | 1.5 | 1.1 | -0.036 | 0.018 | 0.044 | 0.318 |
| TG_16_0_38_2_ | 4.1 | 2.8 | 3.5 | 1.9 | -0.044 | 0.022 | 0.045 | 0.318 |

Only the metabolites with an unadjusted *P* < 0.05 are reported. Data are presented as median with interquartile range (IQR). Differences were tested using linear mixed models with Center and Sex were used as random variables and Phenotype, Age, Bristol Stool Score, Habitual Energy Intake, and Habitual Fiber Intake were used as fixed variables. Retrieved P-values were adjusted using Benjamini-Hochberg FDR. FDR-adjusted *Q* < 0.1. Cer, ceramide; C, acylcarnitine; DG, diglyceride; PC, phosphatidylcholine; SM, sphingomyelin; TG, triglyceride.

# Table S5. Network analysis of correlations between microbial taxa at genus level and plasma metabolites. Related to Figure 4C-D.

| **LIR** | | | **MIR** | | |
| --- | --- | --- | --- | --- | --- |
| **Genus** | **Individual correlations w.**  **metabolites (n)** | **Hub ID** | **Genus** | **Individual correlations w.**  **metabolites (n)** | **Hub ID** |
| *G. Alistipes* | 150 | T1 | *G. Terrisporobacter* | 31 | T6 |
| *G. Holdemania* | 90 | T2 | *O. Oscillospirales 010* | 22 | T7 |
| *G. Bilophila* | 63 | T3 | *G. Faecalitalea* | 19 | T8 |
| *G. Bacteroides* | 49 | T4 | *F. Oscillospiraceae 003* | 19 | T9 |
| *G. Barnesiella* | 40 | T5 | *G. Turicibacter* | 17 | T11 |
| *O. Rhodospirillales* | 18 | T10 | *G. Holdemania* | 14 | T2 |
| *G.*  *Catenibacterium* | 12 | T12 | *G. Family XIII AD3011*  *group* | 12 | T13 |
| *G. Bifidobacterium* | 11 | T15 | *F. Ruminococcaceae g1* | 12 | T14 |
| *G. Eubacterium*  *hallii group* | 10 | T18 | *G. Bilophila* | 12 | T3 |
| *G. Adlercreutzia* | 10 | T19 | *C. Bacilli RF39* | 11 | T16 |
| *G. Slackia* | 10 | T20 | *G. Eubacterium*  *coprostanoligenes group* | 11 | T17 |
| *G. TM7x* | 8 | T26 | *G. Izemoplasmatales* | 10 | T21 |
| *G. Odoribacter* | 7 | T27 | *G. Lachnospiraceae UCG*  *004* | 9 | T22 |
| *G. Prevotella* | 6 | T29 | *G. Eubacterium siraeum*  *group* | 9 | T23 |
| *C. Clostridia g1* | 6 | T30 | *F. Oscillospiraceae group* | 9 | T24 |
| *G. Desulfovibrio* | 5 | T33 | *G. Enterobacter* | 9 | T25 |
| *G. Clostridia vadinBB60 group* | 5 | T34 | *G. Eubacterium hallii group* | 8 | T18 |
|  |  |  | *F. Oscillospiraceae 005* | 7 | T28 |
|  |  |  | *F. Prevotellaceae g2* | 6 | T31 |
|  |  |  | *G. Lachnospiraceae*  *NK4A136 group* | 6 | T32 |
|  |  |  | *G. Clostridia vadinBB60*  *group* | 5 | T34 |
|  |  |  | *G. Blautia* | 5 | T35 |
|  |  |  | *G. Flavonifractor* | 5 | T36 |
|  |  |  | *F. Atopobiaceae g1* | 5 | T37 |
|  |  |  | *G. Tyzzerella* | 5 | T38 |

The table indicates all correlations that meet criteria Spearman correlation coefficient > 0.25 and unadjusted P < 0.005. Microbial taxa at genus level are indicated as primary hubs in the network visualization. Only taxa with ≥ 5 correlations that meet the selection criteria are shown.

## Table S6. Participant characteristics of MIR and LIR phenotypes compared to a ‘No IR’ group. Related to Figure 5.

|  | **No IR**  **phenotype** | **LIR**  **phenotype** | **MIR**  **phenotype** | ***P-*value** | ***P*-value** | ***P*-value** |
| --- | --- | --- | --- | --- | --- | --- |
|  | n = 30 | n = 89 | n = 144 | Overall | No IR vs.  LIR | No IR vs MIR |
| **General characteristics** |  |  |  |  |  |  |
| Age, years | 48.7 ± 12.6 | 59.6 ± 7.3 | 60.6 ± 8.1 | **<0.001** | **<0.001** | **<0.001** |
| Women, n (%) | 22 (73.3%) | 46 (51.7%) | 90 (62.5%) | 0.076 | 0.110 | 0.814 |
| Systolic blood pressure,  mmHg | 122 ± 8 | 127 ± 16 | 126 ± 14 | 0.275 | 0.348 | 0.454 |
| Diastolic blood pressure,  mmHg | 82 ± 7 | 80 ± 11 | 79 ± 10 | 0.315 | 0.826 | 0.393 |
| **Anthropometrics and body composition** |  |  |  |  |  |  |
| BMI, kg/m^2^ | 29.9 ± 3.8 | 29.7 ± 3.6 | 29.5 ± 3.2 | 0.082 | 1.000 | 1.000 |
| Waist circumference, cm | 98.2 ± 11.6 | 103.9 ±  10.8 | 101.0 ± 8.8 | **0.014** | **0.022** | 0.467 |
| Waist-to-hip ratio | 0.90 ± 0.07 | 0.95 ± 0.09 | 0.93 ± 0.09 | 0.067 | 0.068 | 0.374 |
| **Glucose homeostasis** |  |  |  |  |  |  |
| Fasting glucose, mmol/L | 5.0 [4.7,  5.5] | 5.7 [5.2,  6.0] | 5.4 [5.1,  5.7] | **<0.001** | **<0.001** | **0.019** |
| 2-hour glucose, mmol/L | 5.4 [4.4,  6.5] | 5.9 [5.2,  7.0] | 7.0 [5.9,  8.4] | **<0.001** | 0.223 | **<0.001** |
| Fasting insulin, pmol/L | 42.4 [30.3,  51.3] | 58.0 [45.2,  75.4] | 52.5 [42.1,  69.5] | **<0.001** | **<0.001** | **0.002** |
| 2-hour insulin, pmol/L | 254.6  [122.4,  317.5] | 374.3  [229.2,  598.3] | 511.1  [334.1,  823.0] | **<0.001** | **0.007** | **<0.001** |
| MISI, AU | 0.196  [0.126,  0.251] | 0.126  [0.106,  0.180] | 0.07  [0.0478,  0.092] | **<0.001** | 0.150 | **<0.001** |
| HIRI, AU | 326.9  [221.2,  394.1] | 626.3  [499.8,  780.4] | 344.0  [271.7,  442.1] | **<0.001** | **<0.001** | 1.000 |

Differences between IR phenotypes were assessed using one-way ANOVA for normally distributed numerical data (mean ± SD) and Kruskal-Wallis tests for non-normally distributed numerical data (median [25^th^ percentile, 75^th^ percentile]). Bonferroni post hoc testing was applied for both methods.

**P* < 0.05 for between groups testing. BMI, body mass index; MISI, muscle insulin sensitivity index; HIRI, hepatic insulin resistance index.


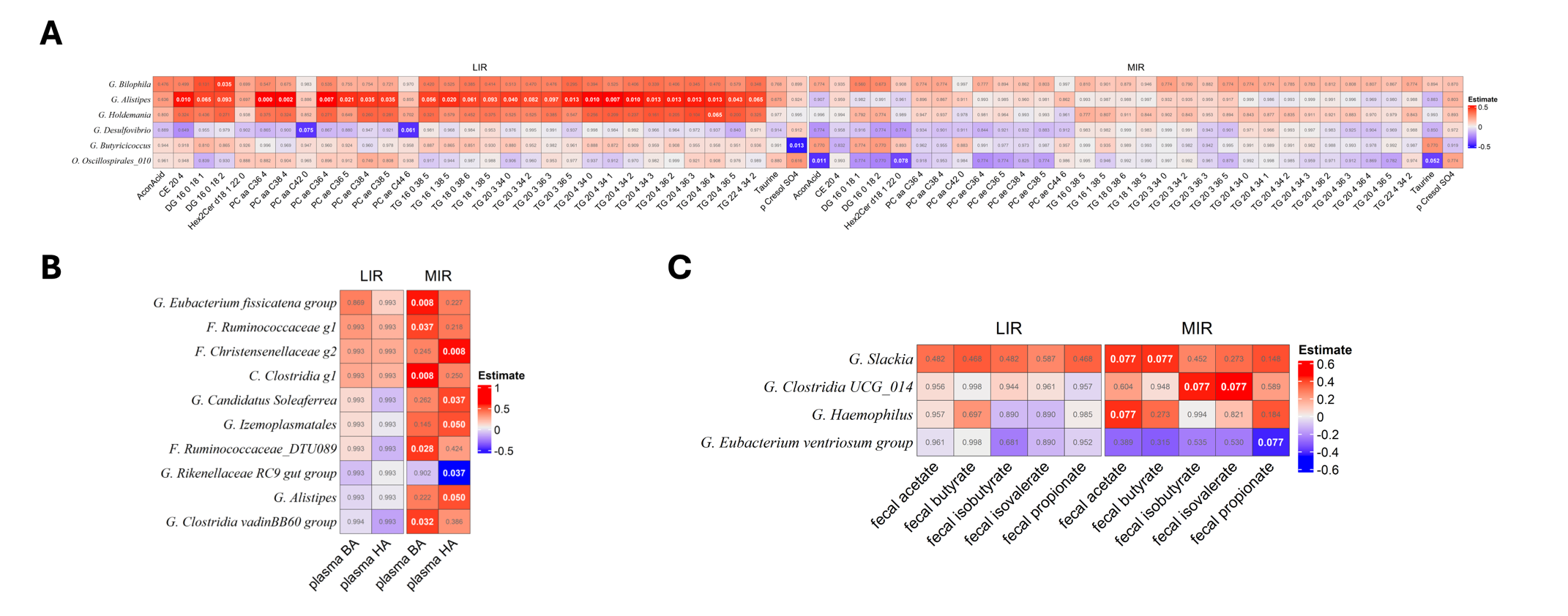


**Figure S1. Correlations of plasma and fecal metabolites with distinct microbial taxa at genus level in LIR and MIR phenotypes. Related to Figure 4.** The heatmaps indicate Spearman’s rank correlation analysis between (A) plasma metabolites (metbolomics), (B) plasma SCFA/BCFA and

(C) fecal SCFA/BCFA. Only correlations that are significant in either MIR or LIR are shown in bold (FDR-adjusted *Q*-value < 0.1). BA, bytyrate; BCFA; branched-chain fatty acid; CE, cholesterol ester; DG, diglyceride; DHA, docosahexaenoic acid; FA, fatty acid; HA, hexanoate (caproate); HexCer, hexosylceramide, LIR, liver insulin resistance; MIR, muscle insulin resistance; PC, phosphatidylcholines; SCFA, short-chain fatty acid; SM, sphingomyelin;TG,triglyceride.

8
